# Associations between serum polybrominated diphenyl ethers and thyroid hormones in a cross sectional study of a remote Alaska Native population

**DOI:** 10.1038/s41598-018-20443-9

**Published:** 2018-02-02

**Authors:** Samuel C. Byrne, Pamela Miller, Samarys Seguinot-Medina, Vi Waghiyi, C. Loren Buck, Frank A. von Hippel, David O. Carpenter

**Affiliations:** 1 Environmental Studies, St. Lawrence University, Canton, NY USA; 2 Alaska Community Action on Toxics, Anchorage, AK USA; 3 Department of Biological Sciences and Center for Bioengineering Innovation, Northern Arizona University, Flagstaff, AZ USA; 4 Institute for Health and the Environment, University at Albany, Rensselaer, NY USA

## Abstract

Polybrominated diphenyl ethers (PBDEs) are ubiquitous environmental pollutants. Arctic indigenous peoples are exposed to PBDEs through a traditional diet high in marine mammals. PBDEs disrupt thyroid homeostasis. The aim of this study was to assess the relationship between serum PBDEs and thyroid function in a remote population of St. Lawrence Island Yupik. Serum samples were collected from 85 individuals from St. Lawrence Island, Alaska and measured for concentrations of PBDEs, free and total thyroxine (T4), free and total triiodothyronine (T3), and thyroid stimulating hormone (TSH). The relationships between PBDEs and thyroid hormones were assessed using multiple linear regression fit with generalized estimating equations. Serum concentrations of several Penta-BDE congeners (BDE-28/33, 47, and 100) were positively associated with concentrations of TSH and free T3, while serum concentration of BDE-153 was negatively associated with total T3 concentrations. Both BDE-47 and 153 remained significantly associated with thyroid hormones when BDE-47, BDE-153, and BDE-209 were covariates in the same model. There were no significant relationships between serum concentrations of PBDEs and either free or total T4. Individual PBDEs are associated with thyroid hormones in serum from a remote population of Alaska Natives, and directions of effect differ by congener.

## Introduction

Polybrominated diphenyl ethers (PBDEs) are additive flame retardants still found in numerous products such as urethane foam, electronics and fabrics. Due to their widespread usage and persistence in the environment, PBDEs have become globally ubiquitous environmental contaminants^1^. In the United States, where most PBDEs were used, they have been voluntarily phased out of production beginning in 2004^2^. All three technical formulations, penta-BDE, octa-BDE, and deca-BDE are globally restricted under the Stockholm Convention on Persistent Organic Pollutants due to evidence of toxicity and bioaccumulation in humans and animals^3^. Despite recently implemented restrictions, the large reservoir of PBDEs in the environment and products ensures continued exposure to these compounds.

Alaska Natives are exposed to PBDEs through two primary sources: diet and household dust. Characteristic of many arctic indigenous peoples, Alaska Natives are heavily reliant on a subsistence diet. Among the Yupik residents of St. Lawrence Island, Alaska this diet includes high trophic level and/or long lived marine mammals such as bowhead whale (*Balaena mysticetus*), Pacific walrus (*Odobenus rosmarus*), and bearded seal (*Erignathus barbatus*). Bioaccumulation of PBDEs in arctic and sub-arctic marine mammals is well documented^4–7^ and thus their dietary consumption represents an important source of PBDE exposure^8,9^. Residents of St. Lawrence Island are also exposed to PBDEs through the indoor environment; we previously reported that concentrations of PBDEs in house dust from St. Lawrence Island were comparable to those found in the contiguous United States^10^. The health implications of this exposure for arctic indigenous peoples are unknown and have been poorly studied.

Numerous toxicological studies suggest that PBDE exposure decreases concentrations of circulating thyroid hormones through multiple mechanisms, including displacement from transport proteins, as well increased conjugation, metabolism and excretion of hormones^11^. However, there is a lack of consistency with regard to the strength and direction of effects reported in epidemiological studies. Some document inverse associations between PBDEs and thyroid hormones, while others report positive associations. A recent prospective study of adult office workers employing repeated measures of both PBDEs and thyroid hormones found significant inverse associations between BDE-47 and 100 and total thyroxine (T4), but no association with thyroid stimulating hormone (TSH)^12^. This is consistent with the hypothyroxinemic effect predicted by most animal studies^13,14^. However, positive associations with thyroid hormones have also been reported. In a study of adult male sportfish consumers, BDE-99 and BDE-153 were positively associated with T4^15^. A study of Inuit adults from Canada reported a positive association between BDE-47 and total triiodothyronine (T3)^16^. Clearly, the relationship between PBDE exposure and thyroid hormones is complex and not fully understood. The aim of the current study was to determine the association between PBDE exposure and thyroid hormones in a remote population of Alaska Natives, including an assessment of potentially differential effects of individual PBDEs on thyroid function.

## Methods

This study was conducted in two Native Villages, Gambell and Savoonga, on St. Lawrence Island. Participants were recruited through flyers posted in public spaces, or directly recruited by bilingual (Yupik-English) community health researchers. Inclusion criteria included being of reproductive age defined as 18–45 years. Community health researchers aimed to recruit one man and one woman from each participating home. A total of 85 individuals from 49 homes were recruited for the study. There were a total of 36 male-female pairs, and an additional 11 unpaired women and 2 unpaired men. This study was approved by the Alaska Area IRB (Indian Health Service IRB00000636) and the Research Ethics Review Board of the Norton Sound Health Corporation. All participants signed an informed consent (administered by bilingual research assistants) before participating in the study. All research was performed in accordance with relevant guidelines and regulations.

Approximately 60 mls of venous blood was drawn into sterile vacutainers (Becton Dickinson, Franklin Lakes, NJ) from individuals who had fasted for at least 8 hours. Blood was allowed to clot at room temperature for one hour, then centrifuged for 15 minutes at 3300 rpm. Serum was then sub-divided for chemical and hormone analyses. For PBDE analysis, serum was transferred to glass vials with PTFE lined caps (Becton Dickinson, Franklin Lakes, NJ), stored at −18 °C, and shipped frozen. Quantification of analytes in serum was carried out by AXYS Analytical (Sydney, British Columbia, Canada). Forty individual PBDEs were quantified using isotope dilution high resolution gas chromatography/high resolution mass spectrometry. Isotopically labeled surrogate standards were used for PBDE analysis. A blank and matrix spike were included in each analytical batch of approximately 13 samples. Analyte recoveries from matrix spikes were never outside of 80–120%. BDE-209 recovery ranged from 60–120%. When blanks contained quantifiable concentrations of analytes, this concentration was subtracted from the other samples in the analytical batch. The limit of detection(LOD) ranged from approximately 0.01 to 1 pg/ml for most congeners, but was higher for BDE-209 ranging from 0.4 to 26 pg/ml. Approximately 2 ml serum aliquots were frozen in the field at −18 °C and shipped overnight to Labcorp (Seattle, Washington) for analysis of thyroid hormones. TSH, T3, free triiodothyronine (fT3), and free thyroxine (fT4) were quantified using an Electro-chemiluminescence immunoassay (ECLIA). Precision was not determined for the samples under study, however method precision is reported as maximum observed coefficients of variation (CV) in human serum reported by Labcorp. T3 had a maximum CV of 5.4%, cross reactivity of <1% for other thyroid hormones, and a LOD of 0.195 ng/mL. fT3 has a maximum CV of 8.2%, cross reactivity of <0.01% for other thyroid hormones, and a LOD of 0.06 ng/dL. fT4 had a maximum CV 7.6%, cross reactivity of ≤0.005% for other thyroid hormones, and a LOD of 0.101 ng/dL. T4 was quantified using a cloned enzyme donor immunoassay (CEDIA) with a maximum CV of 9.2%, cross reactivity of <0.1% for other thyroid hormones, and a LOD of 0.5–20 μg/dL. Total serum lipids were determined by enzymatic measurements of cholesterol, free cholesterol, triglycerides and phospholipids^17^.

TSH as well as all PBDEs were natural log transformed to better fit a normal distribution. Spearman’s rank correlation was used to assess correlation among PBDEs. PBDE concentrations are reported as percentiles. The associations between PBDEs and circulating thyroid hormones were estimated using multiple linear regression estimated with generalized estimating equations (GEE). GEE was used to account for the correlation of multiple participants from the same home. The wet weight concentrations of individual PBDEs were used as the predictor variables in each of five models with T3, fT3, T4, fT4 and TSH as the dependent variables, adjusting for age, sex, smoking, and serum lipid content. PBDE concentrations were lipid adjusted within the model rather than lipid standardized^18^. Regression coefficients represent the predicted change in thyroid hormones for a one intra-quartile range increase in PBDE concentration, controlling for covariates. Confounders were identified a priori using a directed acyclic graph^19^. Additionally, a model was constructed using multiple individual PBDEs as covariates in a single model, for each of five thyroid measures, and adjusted for the same confounders. This model used only BDE-47, BDE-153 and BDE-209. These PBDEs were chosen for several reasons: 1) they had high detection rates and were present at relatively high concentrations, 2) BDE 47 and 209 are good indicators of their respective technical mixtures, 3) BDE 47, 153, and 209 are minimally correlated. For all models, regression coefficients represent the effect of a one intraquartile range (IQR) change in PBDE concentration. Because machine read values were not available, PBDE concentrations that were < LOD were imputed as LOD/√2 for regression analysis^20^. A sensitivity analysis was conducted in which continuous PBDEs were also modeled with data < LOD imputed as the mean of observed values, a method which produces minimally biased conservative estimates^21^. The results of these two methods were compared to ensure data < LOD were having a minimal impact on regression estimates (supplemental material). Influential observations were identified using DFBETAS which is a standardized measure of the impact of a single observation on the regression coefficient; in the event a DFBETAS was >1 the observation was removed from the regression, and the regression was re-run. Statistical analysis was conducted in SAS 9.4 (SAS Institute, Cary, NC).

## Results

Table 1 presents descriptive statistics for participant characteristics. The median age of participants was 29 years. Age ranges of men and women in the study were comparable (19–45 in men, and 18–45 in women). Tobacco smoke exposure, measured as active smoking or the presence of an active indoor smoker in the home, was slightly more prevalent in women (17%) than men (13%). Detection rates for PBDEs were similar among men and women, but slightly higher in women for BDE-47, BDE-100, and BDE-209. Median concentrations tended to be similar in men and women. A female participant with elevated serum PBDE concentrations skewed the distributions in females, specifically for BDEs 47, 99 and 100. Maximum PBDE concentrations were uniformly higher among women (Table 1). No individuals reported current or former thyroid disease, or current thyroid medication. One individual had a TSH concentration above the reference range. Eight individuals had TSH concentrations below the reference range. One individual had both T3 and fT3 concentrations above the reference ranges. Two individuals had T4 concentrations above the reference range, and one had T4 below the reference range. Unless specified all other hormones in these individuals were within the reference ranges.

**Table 1:** Descriptive statistics for study participants.

	Men (n = 38)			Women (n = 47)		Laboratory reference range
	median	range		median	range	
TSH (µIU/ml)	1.28	0.32–4.65		0.96	0.05–4.47	0.45–4.5
T4 (µg/dL)	9.0	4.8–13.8		8.1	4.3–11.8	4.5–12
fT4 (ng/dL)	1.30	0.95–1.71		1.23	0.94–1.67	0.82–1.77
T3 (pg/mL)	143	109–176		137	92–197	71–180
fT3 (ng/dL)	3.6	2.8–4.4		3.2	2.2–4.5	2–4.4
Age (yr)	29	19–45		28	18–45	
Smoke^a^ n(%)	5 (13%)			8 (17%)		
**PBDEs pg/ml ww**	**median**	**range**	**% detect**	**median**	**range**	**% detect**
BDE-28/33	3.22	0.37–25.05	100	4.17	0.49–48.65	100
BDE-47	46.57	nd-463.94	97	60.34	0.80–2538.94	100
BDE-99	9.19	nd-60.03	89	9.71	nd-831.31	89
BDE-100	9.96	nd-93.94	97	11.94	nd-360.84	98
BDE-153	59.64	20.31–180.91	100	43.21	3.18–251.87	100
BDE-209	18.39	nd-204.22	97	17.87	0.33–283.35	100

^a^= active smoking in home; nd = non-detect.

Several PBDE congeners were positively associated with serum concentrations of TSH (Table 2). A one IQR increase in BDE-28/33 was positively associated with TSH (β = 0.41; 95% confidence interval (CI) 0.19, 0.63; p < 001), as were BDE 47 (β = 3.87; 95% CI 1.21, 6.57; p < 0.005) and BDE-100 (β = 0.89; 95% CI 0.18, 1.61; p = 0.01).

**Table 2:** Associations between individual PBDEs and thyroid hormones controlling for age, sex, smoking, and serum lipids from multiple linear regression estimated with GEE.

(ln)TSH					Total T4					Free T4				
	**β 95% CI**			**p-value**		**β 95% CI**			**p-value**		**β 95% CI**			**p-value**
**BDE_28/33**	0.41	0.19	0.63	** < 0.001**	**BDE_28/33**	−0.48	−1.46	0.50	0.34	**BDE_28/33**	−0.02	−0.08	0.05	0.62
**BDE-47**	3.87	1.21	6.57	** < 0.005**	**BDE-47**	−7.18	−16.60	2.23	0.13	**BDE-47**	0.13	−0.64	0.90	0.75
**BDE-99**	0.20	−0.17	0.57	0.30	**BDE-99**	1.05	0.00	2.11	0.05	**BDE-99**	0.00	−0.07	0.08	0.92
**BD-100**	0.89	0.18	1.61	**0.01**	**BD-100**	0.06	−2.18	2.30	0.96	**BD-100**	−0.08	−0.23	0.07	0.31
**BDE-153**	−0.08	−5.44	5.27	0.98	**BDE-153**	1.27	−13.59	16.13	0.87	**BDE-153**	0.02	−0.95	0.98	0.97
**BDE-209**	0.02	−0.16	0.21	0.81	**BDE-209**	0.43	−0.27	1.12	0.23	**BDE-209**	0.04	−0.01	0.09	0.11
**Total T3**					**Free T3**									
	**β 95% CI**			**p-value**		**β 95% CI**			**p-value**					
**BDE_28/33**	2.51	−4.68	9.70	0.49	**BDE_28/33**	0.18	0.07	0.30	** < 0.005**					
**BDE-47**	30.84	−34.33	96.04	0.35	**BDE-47**	2.21	0.46	3.95	**0.01**					
**BDE-99**	6.52	−4.36	17.39	0.24	**BDE-99**	0.15	−0.01	0.31	0.07					
**BDE-100**	3.11	−13.95	20.17	0.72	**BD-100**	0.39	0.12	0.66	** < 0.005**					
**BDE-153**	−113.14	−225.04	−1.14	**0.05**	**BDE-153**	−1.76	−4.36	0.85	0.19					
**BDE-209**	−1.27	−7.01	4.47	0.66	**BDE-209**	0.04	−0.08	0.15	0.53					

When modeled individually, no PBDEs were associated with serum concentrations of either f T4 or T4 (Table 2). BDE-99 was marginally associated with T4 after the removal of an influential data point with a high BDE-99 concentration.

A one IQR change in BDE-153 was negatively associated with serum concentrations of T3 (Table 2) (β = −113.14; 95% CI –225.04, −1.14; p = 0.048). Several penta-BDE congeners were positively associated with fT3 concentration. BDE-28/33 was positively associated with fT3 (β = 0.18; 95% CI 0.07, 0.30; p < 0.005), as was BDE-47 (β = 0.2.21; 95% CI 0.46, 3.95; p = 0.01), and BDE-100 (β = 0.39; 95% CI 0.12 0.66; p = 0.005). BDE-209 was not significantly associated with any thyroid hormones.

Table 3 presents the results of models which include a BDE*sex product term to assess effect modification by sex. The table includes the effect of the PBDE in men as well as the joint effect of the PBDE and female sex. The association between BDE-99 and TSH was significantly modified by sex, with a positive association in men (β = 0.75; 95% CI 0.20, 1.30; p = 0.01) and a negative joint effect of BDE-99 and female sex (β = −0.92 95%; CI −1.59, −0.25; p = 0.01). When an influential data point with a high BDE-47 value was removed, sex significantly modified the effect of BDE-47 on T4 (p < 0.001), but this effect was not significant in the full dataset (p = 0.29). Sex also modified the effect of BDE-47 (p = 0.002) and BDE-99 (p = 0.02) on fT4, with a positive effect in women and a negative effect in men. However, no effect modification was detected for any association in the sensitivity analysis (Table S-2).

**Table 3:** Effect estimates for individual PBDEs in males, and joint effect of PBDEs and female sex controlling for age, smoking, and serum lipids, from multiple linear regression estimated with GEE.

PBDE effect among males					PBDE*female joint effect				
	**β 95% CI**			p-value		**β 95% CI**			**p-value**
**TSH**									
**BDE-28/33**	0.53	0.20	0.87	<0.0001	BDE−28/33	−0.26	−0.84	0.33	0.39
**BDE-47**	4.98	1.95	8.01	<0.0001	**BDE-47**	0.59	−5.57	6.72	0.85
**BDE-99**	0.75	0.20	1.30	0.01	**BDE-99**	−0.92	−1.59	−0.25	**0.01**
**BDE-100**	1.43	0.59	2.28	<0.0001	**BDE-100**	−0.93	−2.28	0.42	0.18
**BDE-153**	−2.49	−22.27	17.31	0.81	**BDE-153**	11.37	−10.62	33.33	0.31
**BDE-209**	0.09	−0.17	0.36	0.48	**BDE-209**	−0.11	−0.48	0.25	0.54
**Total T4**									
**BDE-28/33**	−0.72	−1.95	0.51	0.25	**BDE-28/33**	0.55	−1.18	2.28	0.54
**BDE-47**	−6.93	−15.04	1.18	0.09	**BDE-47**	18.88	8.47	29.33	** < 0.0001**
**BDE-99**	−0.01	−1.69	1.67	0.99	**BDE-99**	0.76	−1.14	2.65	0.44
**BDE-100**	−2.54	−5.36	0.27	0.08	**BDE-100**	2.75	−0.66	6.17	0.11
**BDE-153**	2.61	−4.57	9.79	0.48	**BDE-153**	−3.88	−12.41	4.63	0.37
**BDE-209**	0.56	−0.74	1.86	0.40	**BDE-209**	−0.19	−1.35	0.98	0.75
**Free T4**									
**BDE-28/33**	−0.07	−0.17	0.03	0.17	**BDE-28/33**	0.12	0.00	0.24	0.06
**BDE-47**	−0.95	−1.62	−0.28	< 0.0001	**BDE-47**	1.46	0.51	2.39	** < 0.0001**
**BDE-99**	−0.04	−0.17	0.09	0.52	**BDE-99**	0.08	−0.07	0.23	0.31
**BDE-100**	−0.24	−0.46	−0.01	0.04	**BDE-100**	0.30	0.05	0.56	**0.02**
**BDE-153**	−1.10	−2.84	0.64	0.22	**BDE-153**	1.70	−0.41	3.82	0.11
**BDE-209**	0.06	−0.02	0.14	0.16	**BDE-209**	−0.02	−0.11	0.07	0.60
**Total T3**									
**BDE−28/33**	4.58	−5.11	14.27	0.35	**BDE-28/33**	−4.19	−21.49	13.10	0.63
**BDE-47**	40.54	−33.10	114.18	0.28	**BDE-47**	64.66	−71.59	201.16	0.35
**BDE-99**	4.29	−11.05	19.64	0.58	**BDE-99**	3.83	−18.53	26.18	0.74
**BDE-100**	14.30	−8.46	37.01	0.22	**BDE-100**	−20.34	−60.14	19.40	0.32
**BDE-153**	−42.07	−224.07	140.12	0.65	**BDE**−**153**	−96.69	−330.22	136.84	0.42
**BDE-209**	1.15	−5.98	8.29	0.75	**BDE**−**209**	−3.76	−12.33	4.81	0.39
**Free T3**									
**BDE-28/33**	0.14	−0.06	0.33	0.17	**BDE-28/33**	0.10	−0.16	0.36	0.44
**BDE-47**	1.64	0.21	3.08	0.02	**BDE-47**	0.90	−1.10	2.90	0.38
**BDE-99**	0.11	−0.09	0.32	0.28	**BDE-99**	0.06	−0.24	0.36	0.69
**BDE-100**	0.48	0.07	0.90	0.02	**BDE-100**	−0.18	−0.75	0.39	0.53
**BDE−153**	−1.76	−4.88	1.35	0.27	**BDE-153**	0.00	−3.59	3.59	0.99
**BDE-209**	0.13	−0.02	0.28	0.10	**BDE-209**	−0.14	−0.32	0.05	0.15

Table 4 presents the results of models that simultaneously modeled BDE-47, BDE-153 and BDE-209 as predictors, in order to assess the potential for confounding by other PBDEs. BDE-47, 153, and 209 were chosen because they are weakly to moderately inter-correlated and therefore could be simultaneously modeled (Table S-3). BDE-47 remained significantly positively associated with TSH (β 4.57; 95% CI 1.90, 7.24; p < 0.001) for every one IQR change PBDE concentration. BDE-47 appeared significantly associated with T4 (β −9.06; 95% CI −17.73, −0.38; p = 0.04). After adjusting for other PDBEs, a one IQR change in BDE-47 was significantly positively associated with T3 (β = 76.08; 95% CI 23.68, 128.47; p = 0.004) and a one IQR change in BDE-153 remained negatively associated with T3 (β = −153.8; 95% CI −268.85, −38.64; p = 0.009). BDE-47 remained significantly positively associated with fT3 (β = 4.16; 95% CI 2.41, 5.90; p < 0.0001) for every one IQR change in PBDE concentration. Including an influential data point with a high BDE-47 value made this association weaker (β = 3.08) but still significant (p = 0.003). A one IQR change in BDE-153 was negatively associated with fT3 after adjustment for other PBDEs (β = −3.94; 95% CI −6.08, −1.81; p < 0.001).

**Table 4:** Associations between individual PBDEs and thyroid hormones controlling for age, sex, smoking, serum lipids, and other PBDEs, from multiple linear regression estimated with GEE.

TSH					Total T4					Free T4				
	**β**	**95% CI**		**p-value**		**β**	**95% CI**		**p-value**		**β**	**95% CI**		**p-value**
**BDE-47**	4.57	1.90	7.24	** < 0.001**	**BDE-47**	−9.06	−17.73	−0.38	**0.04**	**BDE-47**	0.08	−0.87	1.00	0.89
**BDE-153**	2.82	−7.97	2.35	0.29	**BDE-153**	2.90	−10.62	16.39	0.67	**BDE-153**	−0.17	−1.27	0.91	0.75
**BDE-209**	0.02	−0.17	0.21	0.86	**BDE-209**	0.50	−0.23	1.23	0.18	**BDE-209**	0.04	−0.01	0.09	0.10
	**Total T3**					**Free T3**								
	**β**	**95% CI**		**p-value**		**β**	**95% CI**		**p-value**					
**BDE-47**	76.08	23.68	128.47	**0.004**	**BDE-47**	4.16	2.41	5.90	** < 0.0001**					
**BDE-153**	−153.80	−268.85	−38.64	**0.01**	**BDE-153**	−3.94	−6.08	−1.81	** < 0.001**					
**BDE-209**	−0.99	−6.91	4.92	0.74	**BDE-209**	0.03	−0.07	0.13	0.58					

## Discussion

In this study of adult Alaska Natives, BDE-28/33, 47, 99, and 100 were individually and positively associated with TSH. BDE-47 was positively associated with TSH after controlling for the effects of BDE-153 and BDE-209. However, BDE-28/33, 47, 99, and 100 have a high mutual correlation, and it is therefore difficult to partition the effects of these compounds. The overall suggestion is that at least one component congener of penta-BDE is positively associated with TSH. While infrequent, other epidemiological studies have reported positive associations between penta-BDE and TSH. A small study of e-waste workers found higher TSH concentrations among the most highly exposed group^22^. In a study of young children from the southeastern United States, the sum of penta-BDE was positively associated with TSH^23^. Multiple PBDEs were positively associated with TSH among late-term pregnant women from California^24^; however, the opposite association has also been reported in pregnant women from California^25^. An inverse association between BDE-47 and TSH was reported in a study of males with high fish intake^26^. Further complicating matters, some studies have reported no association between PBDEs and TSH^16,27^. There is minimal evidence for PBDEs causing secondary hyperthyroidism, suggesting that any positive associations with TSH may be the result of adaptive TSH increases due to sub-clinical hypothyroidism. However, the hypothyroxinemic effect seen in animal models^14^ suggests that PBDEs may disrupt thyroid homeostasis in ways other than classic hyperthyroid or hypothyroid effects. In addition, caution should be exercised in inferring physiological explanations for statistical relationships, given the apparent complexity of these relationships.

In the current study we did not find any associations between individual PBDEs and fT4 or T4 when they were modeled individually. Epidemiological studies of adults have documented both inverse associations^12^ and positive associations^15^ between PBDEs and T4. In our study of Alaska Natives, removal of a participant with high serum BDE-47 concentration from the models resulted in an apparent departure from additivity of the effect of BDE-47 and female sex on T4. The interaction predicted a significant inverse association in men and positive association in women. However, the relationship was not significant in the sensitivity analysis, thus we cannot rule out the possibility that the interaction resulted from the handling of data <LOD. To the best of our knowledge, effect modification of the BDE-47 and T4 association by sex has not been previously reported. Overall, the results of this study do not suggest a strong interaction between sex and PBDE exposure on thyroid hormone homeostasis. A negative association between BDE-47 and T4 was detected when BDE-153 and BDE-209 were included in the same model.

Several PBDEs were positively associated with fT3 in this population, including BDE-28/33, BDE-47, BDE-100, and a marginally significant association for BDE-99. In contrast, BDE-153 was significantly negatively associated with T3 among participants of this study. No interactive terms were significant for fT3 or T3 models. When BDE-47, 153, and 209 were modeled together, both BDE-47 and BDE-153 were significantly associated with fT3 and T3. Among a large sample of Inuit adults from Canada, serum BDE-47 concentrations were positively associated with T3, however fT3 was not measured^16^. BDE-153 was also measured, but was not associated with any thyroid hormones among the Canadian Inuit. A study of young children in Atlanta also reported positive associations between penta-BDE congeners and fT3^23^. In a study of adult male sportfish consumers, BDE-47, 99, 100, and 153 were positively associated with reverse T3 (rT3), but BDE-47 and BDE-153 were negatively associated with T3^15^. A non-significant inverse association between BDE-153 and T3 was reported in a cohort study utilizing repeated measures of both exposure and thyroid hormones^12^. An inverse association between BDE-153 and T3 was also reported in an epidemiological study of pregnant women^28^. A similar relationship has been reported in cord blood^29^. However, the generalizability of associations found in pregnant women remains unclear, due to estrogen related changes in thyroid homeostasis^30^.

The mechanisms which may underlie these associations are not clear. Associations between PBDEs and T3 or rT3 may suggest alterations of deiodinase concentrations or activity. In our study, positive associations with T3 may suggest an increase in deiodination of T4. In human liver cells, both BDE-99 and BDE-209 induce type I deiodinase expression^31^. However, the opposite effect has been observed. Human glial cells exposed to PBDEs show decreases in type II deiodinase activity^32^. Research in neonatal rats has also found transient decreases in type I deiodinase expression^33^. Displacement of T4 from binding proteins has been suggested as a mechanism of PBDE action^11^. An increase in fT4 could also result in positive associations with T3 if it was efficiently deiodinated into T3. Differences in effects based on species, tissue or individual PBDEs are possible.

Several studies have suggested that the associations between PBDEs and the thyroid hormone axis are modified by other factors. For example, among National Health and Nutrition Examination Survey (NHANES) participants, PBDEs are more strongly associated with thyroid disease in post-menopausal women than in other women^34^, a result that suggests interplay involving the reproductive axis. Immune function may also modify or mediate the relationship between PBDEs and thyroid homeostasis, as differential effects have been reported in individuals with antibodies to thyroglobulin or thyroperoxidase^35^. Some authors have speculated that the exposure response relationship between PBDEs and thyroid hormones is non-monotonic^36^. The hormetic effects of PBDE exposure have been documented in relation to proliferation of cancer cells^37,38^. Hydroxylated PBDEs (OH-BDEs) were not measured as part of this study, though they may play a role in PBDE related thyroid disruption. Specifically, OH-BDEs are structurally similar to thyroid hormones, and bind to thyroid transport proteins with greater affinity than PBDEs, and OH-BDEs bind the thyroid receptor with different effects (agonist/antagonist) dependent on level of bromination^39–43^. Collectively, the literature suggests a complex relationship, potentially modified by numerous factors such as age, sex, immune status, iodine sufficiency and exposure to other environmental chemicals. Our results suggest that individual PBDEs may affect thyroid hormone concentrations differently and that different mechanisms or combinations of mechanisms may be at work.

A few individuals had thyroid hormone concentrations outside the laboratory reference ranges, but none showed characteristic patterns associated with disease. For example, several individuals had low TSH with T4 and T3 in the reference range, which could represent sub-clinical hyperthyroidism. Unfortunately, there is great variation in circulating thyroid concentrations in apparently euthyroid individuals and references ranges are inconsistent across studies^44–47^. In the absence of more detailed clinical information, it is not possible to identify clinical thyroid disease. While this study cannot make inferences about clinically diagnosed thyroid disease, variation in thyroid hormones in the absence of thyroid disease is associated with morbidities. For example, subclinical hypothyroidism is associated with overweight and obesity^48^, adverse cardiovascular outcomes^49,50^, and impaired cognitive function^51^. Subclinical hyperthyroidism is associated with osteoporosis and abnormal cardiac morphology and function^52^.

### Limitations

The cross-sectional design of this study did not allow an examination of the temporal relationship between exposure and outcome. However, the serum concentration of PBDEs is thought to be an indicator of both past and current exposure, due to relatively long biological half-lives^53,54^. This was a convenience sample and may not be representative of the population of St. Lawrence Island. However, it is unlikely that participation rates were differential with regard to thyroid function, making any source of bias small for these associations. Given that no participants reported clinically diagnosed thyroid disease, this study cannot provide insight into the role of PBDEs in thyroid disease. The reported prevalence of smoking is low considering the anecdotally high tobacco use in this population. The small sample size limits the statistical power of the study; low power complicates interpretation of null findings, as they may be the result of type II error. PBDEs, especially those found within the same technical mixtures, are often highly correlated in serum samples^55^. The high mutual correlations among PBDEs make attributing health outcomes to a single compound difficult. BDE-209 is subject to several sources of measurement error, including high risk of sample contamination from the environment, and degradation during analysis^56^. This non-differential error would bias associations toward the null. There is debate about the role of overweight and obesity in thyroid physiology^57^. If it is the case that alteration in body mass index (BMI) is secondary to thyroid disruption, then it is not appropriate to include BMI in the models; however, BMI may also alter thyroid physiology. We did not control for BMI in the models. While participants reported fasting for >8 hours, it is possible that length of fast could impact thyroid hormone concentrations, especially if there was poor compliance. Measures of liver function, and specifically liver deiodinase activity, could provide insight into mechanisms of PBDE effects, but were not collected in this study. This study does not correct for multiple statistical tests^58^, which could increase the probability finding false positive associations.

The residents of St. Lawrence Island are exposed to numerous persistent organic pollutants (POPs) through their traditional diet^59^; and they have higher levels of certain POPs, including polychlorinated biphenyls (PCBs), than populations of the contiguous United States (Carpenter *et al*. 2005). The correlation of PBDEs with other POPs may affect the associations found in this study. Associations between PCBs and thyroid hormones have been reported in both epidemiological studies and in animal models^60,61^. Concurrent exposure to PBDEs and PCBs may have additive or synergistic effects on T4 concentrations^62,63^. In a study of older adults, PBDEs and PCBs were found to synergistically increase the concentration of serum T3 in women^64^. Therefore, there may be confounding or interactions with other POPs that were not captured in this study.

Sensitivity analysis suggests that given a small percentage of missing data, the value chosen for data <LOD has a small impact on effect estimates. Given relatively small percentages of missing data, simple imputation can produce minimally biased effect estimates^21^; however, simple imputation may poorly approximate the variance. This may impact estimates of the standard error and artificially reduce p-values. The empirical (i.e. robust/sandwich) estimator used by GEE provides a reasonable estimate of covariance given an unknown covariance structure^65^, but may underestimate the true standard error.

## Conclusions

Serum concentrations of select PBDEs were associated with serum concentrations of thyroid hormones in this population of Alaska Natives. Several component congeners of the penta-BDE mixture were positively associated with TSH and fT3, while BDE-153 was negatively associated with T3. There was not strong evidence of effect modification by sex. Individual PBDEs appear to have independent, and at times opposite statistical relationships with circulating thyroid hormone concentrations. This may suggest complex and perhaps competing mechanisms of toxicity. No participants reported clinically diagnosed thyroid disease, however sub-clinical alterations in thyroid hormones may have implications for health. The role of PBDE exposure in disruption of thyroid hormone homeostasis, and the clinical significance in adults, remain unclear.

## Declarations

### Ethics approval and consent to participate

This Study was approved by the Alaska Area IRB (Indian Health Service IRB00000636) and the Research Ethics Review Board of the Norton Sound Health Corporation. All participants provided an informed consent (administered by bilingual research assistants) before participating in the study.

### Availability of data and material

The data are not publicly available due to them containing information that could compromise research participant privacy and consent. Data may be available from the authors upon reasonable request and with permission of the St. Lawrence Island working group.

## Electronic supplementary material

supplemental tables
