# Supplementary material for: Associations between serum polybrominated diphenyl ethers and thyroid hormones in a cross sectional study of a remote Alaska Native population
Source: Sci Rep. 2018 Feb 2;8:2198. doi: 10.1038/s41598-018-20443-9 (PMC5797183; doi:10.1038/s41598-018-20443-9)
Supplement: Supplementary file 1 — supplemental tables [file 41598_2018_20443_MOESM1_ESM.doc]

# **Associations between serum polybrominated diphenyl ethers and thyroid hormones in a cross sectional study of a remote Alaska Native population**

**Samuel C. Byrne^1^*, Pamela Miller^2^, Samarys Seguinot-Medina^2^, Vi Waghiyi^2^, C. Loren Buck^3^, Frank A. von Hippel^3^, David O. Carpenter^4^**

**1 Environmental Studies, St. Lawrence University, Canton, NY, USA.** [**sbyrne@stlawu.edu**](mailto:sbyrne@stlawu.edu)

**2 Alaska Community Action on Toxics, Anchorage, AK, USA**

**3 Department of Biological Sciences, Northern Arizona University, Flagstaff, AZ, USA**

**4 Institute for Health and the Environment, University at Albany, Rensselaer, NY, USA**

Supplemental data

Table S-1: Associations between individual PBDEs and thyroid hormones controlling for age, sex, smoking, and serum lipids (<LOD=mean of observed)

| **(ln)TSH** |  |  |  |  | **Total T4** |  |  |  |  | **Free T4** |  |  |  |  |
| --- | --- | --- | --- | --- | --- | --- | --- | --- | --- | --- | --- | --- | --- | --- |
| **ß (95% CI)** | |  |  | **p-value** | **ß (95% CI)** | |  |  | **p-value** | **ß (95% CI)** | |  |  | **p-value** |
| **BDE_28/33** | 0.41 | 0.19 | 0.63 | **<0.001** | **BDE_28/33** | -0.48 | -1.46 | 0.50 | 0.34 | **BDE_28/33** | -0.02 | -0.08 | 0.05 | 0.62 |
| **BDE-47** | 6.23 | 2.95 | 9.49 | **<0.005** | **BDE-47** | 7.80 | -2.41 | 18.01 | 0.13 | **BDE-47** | 0.38 | -0.54 | 1.28 | 0.42 |
| **BDE-99** | 0.84 | 0.41 | 1.28 | **<0.001** | **BDE-99** | 0.47 | -1.28 | 2.21 | 0.6 | **BDE-99** | -0.01 | -0.12 | 0.10 | 0.84 |
| **BD-100** | 1.26 | 0.46 | 2.07 | **<0.005** | **BD-100** | 0.90 | -1.85 | 3.65 | 0.52 | **BD-100** | -0.03 | -0.22 | 0.16 | 0.77 |
| **BDE-153** | -0.08 | -5.44 | 5.27 | 0.98 | **BDE-153** | 1.27 | -13.59 | 16.13 | 0.87 | **BDE-153** | 0.02 | -0.95 | 0.98 | 0.97 |
| **BDE-209** | 0.03 | -0.15 | 0.22 | 0.74 | **BDE-209** | 0.44 | -0.25 | 1.13 | 0.21 | **BDE-209** | 0.04 | -0.01 | 0.09 | 0.11 |
|  |  |  |  |  |  |  |  |  |  |  |  |  |  |  |
| **Total T3** |  |  |  |  | **Free T3** |  |  |  |  |  |  |  |  |  |
| **ß (95% CI)** | |  |  | **p-value** | **ß (95% CI)** | |  |  | **p-value** |  |  |  |  |  |
| **BDE_28/33** | 2.51 | -4.68 | 9.70 | 0.49 | **BDE_28/33** | 0.18 | 0.07 | 0.30 | **<0.005** |  |  |  |  |  |
| **BDE-47** | 92.32 | 18.58 | 166.08 | **0.01** | **BDE-47** | 2.54 | 1.08 | 4.03 | **<0.001** |  |  |  |  |  |
| **BDE-99** | -8.88 | -23.68 | 5.92 | 0.24 | **BDE-99** | -0.13 | -0.43 | 0.16 | 0.37 |  |  |  |  |  |
| **BD-100** | -3.55 | -25.64 | 18.54 | 0.75 | **BD-100** | 0.39 | 0.00 | 0.78 | **0.05** |  |  |  |  |  |
| **BDE-153** | -113.06 | -225.04 | -1.04 | 0.05 | **BDE-153** | -1.76 | -4.36 | 0.85 | 0.19 |  |  |  |  |  |
| **BDE-209** | -1.10 | -6.87 | 4.68 | 0.71 | **BDE-209** | 0.04 | -0.07 | 0.15 | 0.49 |  |  |  |  |  |

***Table S-2: Effect estimates for individual PBDEs in males and joint effect of PBDEs and female sex (<LOD=me***an of observed)

|  | **Men** |  |  |  |  | **PBDE*female joint effect** | | |  |
| --- | --- | --- | --- | --- | --- | --- | --- | --- | --- |
|  | **β** | **95% CI** |  | **p-value** |  | **β** | **95% CI** |  | **p-value** |
|  |  |  |  | **TSH** | |  |  |  |  |
| **BDE-28/33** | 0.53 | 0.20 | 0.87 | 0 |  | -0.26 | -0.84 | 0.33 | 0.39 |
| **BDE-47** | 7.44 | 1.05 | 13.80 | 0.02 |  | -5.41 | -14.19 | 3.36 | 0.23 |
| **BDE-99** | 0.82 | 0.01 | 1.62 | 0.05 |  | 0.04 | -0.94 | 1.02 | 0.93 |
| **BD-100** | 1.32 | -0.10 | 2.75 | 0.07 |  | -0.11 | -2.02 | 1.81 | 0.91 |
| **BDE-153** | 2.61 | -4.57 | 9.79 | 0.48 |  | -3.88 | -12.41 | 4.63 | 0.37 |
| **BDE-209** | 0.12 | -0.15 | 0.39 | 0.38 |  | -0.14 | -0.51 | 0.23 | 0.45 |
|  |  |  |  |  |  |  |  |  |  |
|  |  |  |  | **Total T4** | |  |  |  |  |
| **BDE-28/33** | -0.72 | -1.95 | 0.51 | 0.25 |  | 0.55 | -1.18 | 2.28 | 0.54 |
| **BDE-47** | -2.33 | -20.78 | 16.11 | 0.8 |  | 18.06 | -3.57 | 39.67 | 0.1 |
| **BDE-99** | -0.20 | -2.60 | 2.20 | 0.87 |  | -0.91 | -3.60 | 1.77 | 0.5 |
| **BD-100** | -0.80 | -4.64 | 3.04 | 0.68 |  | 3.79 | -1.19 | 8.77 | 0.14 |
| **BDE-153** | -2.49 | -22.27 | 17.31 | 0.81 |  | 11.37 | -10.62 | 33.33 | 0.31 |
| **BDE-209** | 0.59 | -0.69 | 1.88 | 0.36 |  | -0.22 | -1.37 | 0.93 | 0.71 |
|  |  |  |  |  |  |  |  |  |  |
|  |  |  |  | **Free T4** | |  |  |  |  |
| **BDE-28/33** | -0.07 | -0.17 | 0.03 | 0.17 |  | 0.12 | 0.00 | 0.24 | 0.06 |
| **BDE-47** | -0.33 | -1.85 | 1.18 | 0.68 |  | 1.10 | -0.64 | 2.85 | 0.22 |
| **BDE-99** | 0.03 | -0.16 | 0.23 | 0.73 |  | -0.07 | -0.29 | 0.15 | 0.53 |
| **BD-100** | -0.08 | -0.39 | 0.23 | 0.61 |  | 0.09 | -0.28 | 0.47 | 0.63 |
| **BDE-153** | -1.10 | -2.84 | 0.64 | 0.22 |  | 1.70 | -0.41 | 3.82 | 0.11 |
| **BDE-209** | 0.06 | -0.02 | 0.14 | 0.16 |  | -0.02 | -0.11 | 0.07 | 0.61 |
|  |  |  |  |  |  |  |  |  |  |
|  |  |  |  | **Total T3** | |  |  |  |  |
| **BDE-28/33** | 4.58 | -5.13 | 14.27 | 0.36 |  | -4.19 | -21.48 | 13.10 | 0.63 |
| **BDE-47** | 75.95 | -80.31 | 232.20 | 0.34 |  | -64.66 | -296.35 | 167.03 | 0.59 |
| **BDE-99** | -4.00 | -28.09 | 20.10 | 0.75 |  | -7.69 | -37.44 | 22.02 | 0.61 |
| **BD-100** | 17.03 | -15.51 | 49.57 | 0.3 |  | -35.75 | -84.27 | 12.72 | 0.15 |
| **BDE-153** | -41.88 | -223.88 | 140.12 | 0.65 |  | -96.69 | -330.22 | 136.84 | 0.42 |
| **BDE-209** | 1.72 | -5.44 | 8.89 | 0.64 |  | -4.35 | -12.88 | 4.18 | 0.32 |
|  |  |  |  |  |  |  |  |  |  |
|  |  |  |  | **Free T3** | |  |  |  |  |
| **BDE-28/33** | 0.14 | -0.06 | 0.33 | 0.17 |  | 0.10 | -0.16 | 0.36 | 0.44 |
| **BDE-47** | 2.69 | -0.36 | 5.72 | 0.08 |  | -0.21 | -3.80 | 3.41 | 0.91 |
| **BDE-99** | -0.04 | -0.45 | 0.37 | 0.86 |  | 0.10 | -0.47 | 0.67 | 0.73 |
| **BD-100** | 0.54 | -0.08 | 1.17 | 0.09 |  | -0.27 | -1.17 | 0.63 | 0.56 |
| **BDE-153** | -1.76 | -4.88 | 1.35 | 0.27 |  | 0.00 | -3.59 | 3.59 | 0.99 |
| **BDE-209** | 0.14 | -0.01 | 0.29 | 0.07 |  | -0.15 | -0.33 | 0.03 | 0.11 |

Table S-3: Spearman’s Correlations for wet weight PBDE concentrations in serum

|  | **BDE 28-33** | **BDE-47** | **BDE-99** | **BDE-100** | **BDE-153** | **BDE-209** |
| --- | --- | --- | --- | --- | --- | --- |
| **BDE 28-33** | 1 | 0.84 | 0.53 | 0.73 | 0.32 | 0.10 |
|  |  | <.0001 | <.0001 | <.0001 | 0.003 | 0.37 |
| **BDE-47** |  | 1 | 0.62 | 0.86 | 0.30 | 0.17 |
|  |  |  | <.0001 | <.0001 | 0.005 | 0.12 |
| **BDE-99** |  |  | 1 | 0.64 | 0.16 | 0.07 |
|  |  |  |  | <.0001 | 0.13 | 0.55 |
| **BDE-100** |  |  |  | 1 | 0.34 | 0.13 |
|  |  |  |  |  | 0.002 | 0.24 |
| **BDE-153** |  |  |  |  | 1 | 0.07 |
|  |  |  |  |  |  | 0.53 |
| **BDE-209** |  |  |  |  |  | 1 |

***Table S‑4: Associations*** between individual PBDEs and thyroid hormones controlling for age, sex, smoking, serum lipids, and other PBDEs (<LOD=mean of observed)

|  | **TSH** |  |  | |  |  | **Total T4** |  |  |  |  | | **Free T4** |  |  |  |
| --- | --- | --- | --- | --- | --- | --- | --- | --- | --- | --- | --- | --- | --- | --- | --- | --- |
|  | **β** | **95% CI** | |  | **p-value** |  | **β** | **95% CI** |  | **p-value** | |  | **β** | **95% CI** |  | **p-value** |
| **BDE-47** | 5.47 | 1.08 | | 9.88 | **0.02** | **BDE-47** | 1.80 | -7.70 | 11.26 | 0.71 | | **BDE-47** | 0.36 | -0.74 | 1.46 | 0.52 |
| **BDE-153** | -3.40 | -8.28 | | 1.49 | 0.17 | **BDE-153** | 3.24 | -10.00 | 16.48 | 0.63 | | **BDE-153** | -0.27 | -1.33 | 0.81 | 0.63 |
| **BDE-209** | 0.01 | -0.19 | | 0.21 | 0.91 | **BDE-209** | 0.52 | -0.22 | 1.26 | 0.17 | | **BDE-209** | 0.04 | -0.01 | 0.09 | 0.11 |
|  | **Total T3** |  | |  |  |  | **Free T3** |  |  |  | |  |  |  |  |  |
|  | **β** | **95% CI** | |  | **p-value** |  | **β** | **95% CI** |  | **p-value** | |  |  |  |  |  |
| **BDE-47** | 97.99 | 7.57 | | 188.41 | **0.03** | **BDE-47** | 3.87 | 2.03 | 5.75 | **<0.0001** | |  |  |  |  |  |
| **BDE-153** | -159.11 | -270.53 | | -47.71 | **0.01** | **BDE-153** | -3.71 | -5.87 | -1.52 | **<0.001** | |  |  |  |  |  |
| **BDE-209** | -1.07 | -7.01 | | 4.87 | 0.72 | **BDE-209** | 0.03 | -0.08 | 0.13 | 0.61 | |  |  |  |  |  |
